# Amyloid precursor protein dosage normalization rescues neurogenesis and Alzheimer’s Disease phenotypes associated with Down Syndrome

**DOI:** 10.18103/mra.2026.0302

**Published:** 2026-06

**Authors:** Deepika Patel, Karen Rakowiecki, Orly Lazarov

**Affiliations:** Department of Anatomy and Cell Biology, University of Illinois at Chicago, Chicago, IL, USA

**Keywords:** Down Syndrome, Amyloid precursor protein, Neurogenesis, Alzheimer’s disease

## Abstract

Down Syndrome (DS) is the most abundant genetic form of mental retardation. It is caused by the triplication of partial or complete human chromosome 21 (HSA21). The molecular mechanisms of DS are not fully understood. Amyloid precursor protein (APP) resides on HSA21 and is triplicated in DS. While it is not thought to be a part of the “Down syndrome Critical Region”, APP plays a role in developmental and post-natal neurogenesis and synaptic plasticity, thus, its triplication may affect cortical development in DS. Further, mutations in APP cause familial Alzheimer’s disease (AD). However, whether APP overdose is sufficient or required for the development of Alzheimer’s disease in DS is not fully elucidated. Here, we addressed the role of APP overdose in neuronal development and AD pathology. Using DS patient-derived induced pluripotent stem cells in which one copy of APP was silenced using CRISPR-Cas9, we examined developmenta neurogenesis, AD-related pathology and the expression levels of genes on HSA21 that are implicated in DS, neurodegeneration and inflammation. Amyloid precursor protein triplication, in DS induced pluripotent stem cells, led to reduced stem cell proliferation, enhanced differentiation into neurons, increased amyloid pathology, and altered protein levels of selected genes on HSA21. Correction of APP gene dosage rescued the altered neurogenesis phenotype and reduced pathological amyloidogenic processing. These data highlight APP dosage as a key regulator of neuronal maturation and AD pathology in DS, suggesting therapeutic value in targeting APP expression to mitigate neurodevelopmental and neurodegenerative features of the disorder.

## Introduction

Down Syndrome (DS) is a genetic disorder that is caused by the triplication of human chromosome 21 (HSA21)^1^. It is manifested by alterations in brain development, leading to brain dysfunction and intellectual disability^2,3^. However, how triplication of HSA21 induces these alterations is not fully understood. Previous attempts to narrow down genes that are essential or sufficient for the development of DS yielded several versions of the “Down syndrome Critical Region”^4^. These studies remain largely inconclusive, in part because they depend on a limited number of epidemiological cases. Amyloid precursor protein (*APP*) resides on HSA21 and is triplicated in DS, but was not thought to be part of the Down syndrome Critical Region.

Amyloid precursor protein is a ubiquitously expressed, single-pass transmembrane protein^5^. A multitude of binding partners and downstream molecular pathways have been identified precluding a discrete role for APP in cellular functions^6–9^. In the nervous system, APP has been implicated in development, neuromuscular junction function, synaptic plasticity, and neuroprotection^10–14^. Notably, previous reports suggested a role of APP in neurogenesis^15–17^, including the regulation of neural progenitor cell proliferation in the post-natal mouse brain^18,19^ and in human progenitors^20^. Moreover, limited studies exist attempting to isolate the effect of increased APP gene copy number on neuronal development in DS from additional genes located on HSA21. In one, transcriptome profiling of induced pluripotent stem cells-derived neuronal cultures at day 65 of differentiation shows that APP gene dosage by itself cannot explain the full spectrum of amyloid and tau pathology as manifested in AD^21^. In the second, siRNA mediated knockdown of APP reduced enlarged, rapidly migrating growth cones observed in DS fetal cells^22^.

Mutations in *APP* cause familial Alzheimer’s disease^23^. A large proportion of DS patients develop Alzheimer’s disease (AD). Approximately 50% to 70% of DS patients experience dementia by the time they reach 60 to 70 years of age^24,25^. Previous studies suggested that *APP* overdose underlies increased β-amyloid (Aβ) production, altered Aβ_42/40_ in neurons and deposition of the pyroglutamate (E3)-containing amyloid aggregates, but conflicting evidence was reported concerning tau-linked AD phenotypes^26,27^. Together, this suggests that further studies are warranted to determine whether *APP* overdose is sufficient or required for the induction of brain malformation or the development of Alzheimer’s disease (AD) in DS.

Here, we addressed the role of *APP* overdose in neuronal development in DS. Using Down Syndrome-derived induced pluripotent stem cells in which one APP allele was silenced using CRISPR/Cas9 [DS *APP(+/+/−)*], we examined neurogenesis and AD-related pathology. We observed that the correction of *APP* dose rescues impairments in proliferation of neural progenitor cells and in differentiation of neural precursors and neurons. In addition to amyloid processing, we show that *APP* overdose increased tau expression and the correction of *APP* rescued this increase. Notably, correction of *APP* dose rescued overdose of other genes on HSA21, suggesting that it acts upstream of these genes. Together, this study suggests that APP overdosage plays a role in altered neuronal development and in amyloidosis.

## Materials and Methods

### HUMAN-INDUCED PLURIPOTENT STEM CELLS CULTURE, NEURAL PROGENITOR CELLS DIFFERENTIATION AND NEURON MATURATION

**Figure 1.**
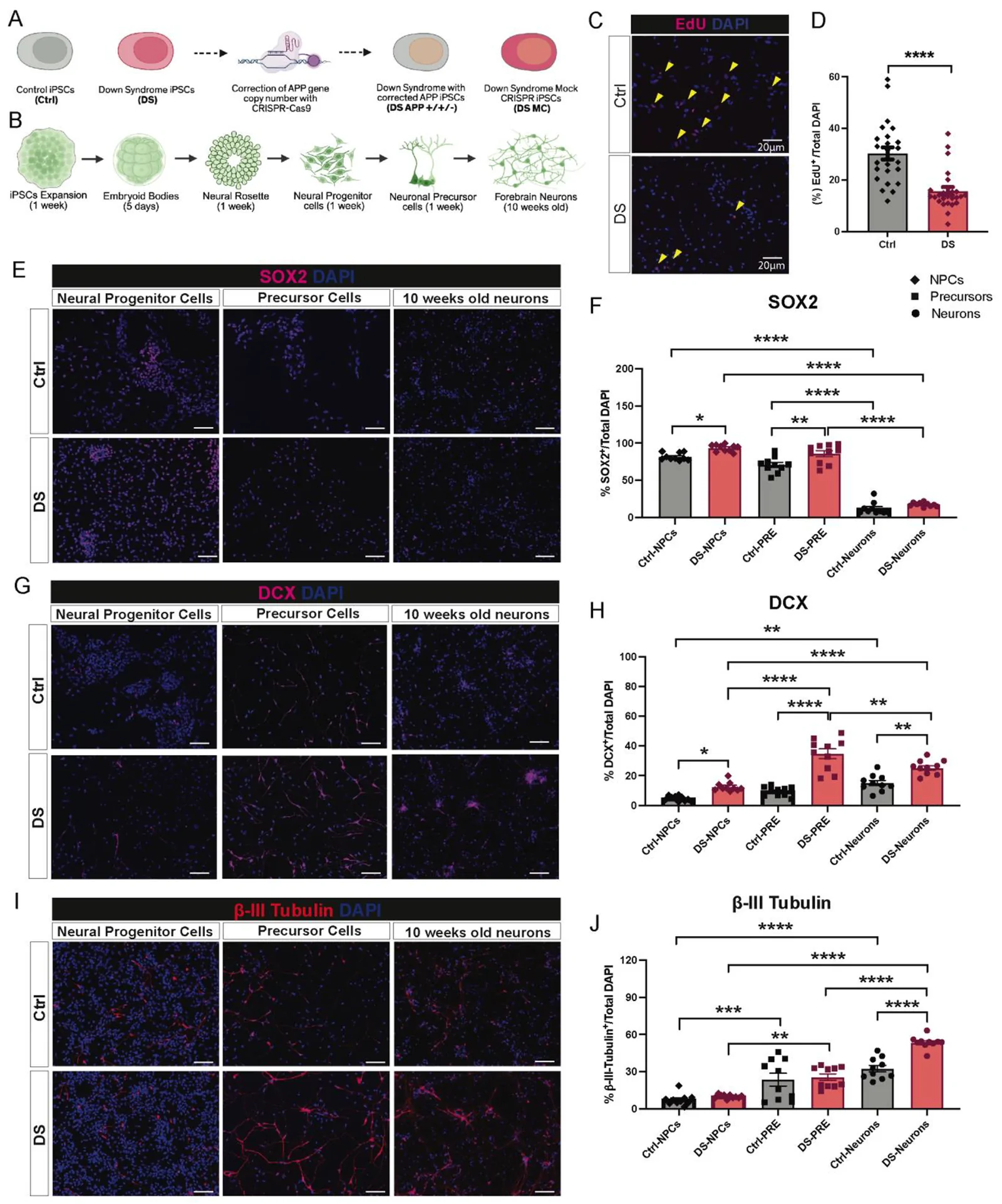
Developmental neurogenesis in control and Down Syndrome induced pluripotent stem cells-derived neural cells. (A) Schematic of patient-derived pluripotent stem cells (iPSCs) experimental groups. Control iPSCs (Ctrl), Down syndrome iPSCs (DS), gene-corrected DS iPSCs (DS APP+/+/−) and a mock CRISPR control (DS MC). (B) Timeline of differentiation of iPSCs to forebrain neurons, indicating the key stages: iPSCs expansion, embryoid body formation, neural rosette induction, neural progenitor cell (NPCs), precursor cells (PRE), and 10-week-old forebrain neurons. (C) Representative EdU immunocytochemistry images showing proliferating (EdU+) neural progenitor cells derived from control and DS iPSCs, and co-stained with DAPI. Yellow arrowheads mark EdU+ cells. Scale bars: 20μm. (D) Quantification of EdU incorporation (%EdU/total DAPI) shows significantly decreased proliferation in DS neural progenitor cells compared to controls. (E, G, I) Representative immunofluorescence images of SOX2 (magenta), DCX (magenta), β-III-tubulin (red) and DAPI (blue) in neural progenitor cells, precursor cells, and 10-week-old neurons from control and DS groups. Scale bars: 20μm. (F, H, J) Quantification of SOX2+, DCX+, and β-III-tubulin+ cells as a percentage of total DAPI+ nuclei across differentiation stages, demonstrates enhanced neuronal differentiation in DS lines. n=9 images per group. Data represented as mean ± SEM with individual points shown. Data analyzed by unpaired t-test (D), two-way ANOVA with Sidak’s multiple comparisons test (F, H, J), *p < 0.05, **p < 0.01, ***p < 0.001, and ****p < 0.0001.

The induced pluripotent stem cells **(iPSCs)** lines used in this study were obtained from repositories and collaborating laboratories. Control line Ctrl #1 (UWWC1-DS2U) and Down syndrome line DS #1 (UWWC1-DS1) were obtained from WiCell. The DS mock-corrected (DS MC) and APP gene-corrected (APP+/+/−) lines were graciously supplied by the Young-Pearse laboratory. The CRISPR targeting of iPSC lines was previously described in^27^. Schematic representation of the experimental groups are depicted in Figure 1A.

Frozen vials from master stocks were thawed at 37°C and plated onto Matrigel hESC-Qualified Matrix-coated plates (Corning, 354277) in mTeSR Plus medium (STEMCELL Technologies, 100-0276), supplemented with 10 μM Y-27632 (ROCK inhibitor) during the first 24 hours post-thaw to enhance survival. The medium was refreshed daily. Individual colonies were mechanically dissected and passaged weekly. Unless otherwise noted, all reagents were purchased from STEMCELL Technologies.

Neural differentiation was performed following STEMCELL Technologies’ protocols, with key stages and validation illustrated in Figure 1B. To generate CNS-type neural progenitor cells (NPCs) from iPSCs, we employed the STEMdiff Neural Induction Medium with SMAD pathway inhibitors using an embryoid body (EB) formation approach. Briefly, confluent iPSCs were dissociated with Gentle Cell Dissociation Reagent and seeded at 3 x 10^6 cells per well into AggreWell 800 plates (preconditioned with Anti-Adherence Rinsing Solution), resulting in approximately 10,000 cells per microwell. The medium was partially replaced daily over five days. The EBs were subsequently collected and plated onto poly-L-ornithine (Millipore sigma, A-004-C)/laminin (Fischer scientific, 23-017-015)-coated 6-well plates to induce neural rosette formation. After 7 days, neural rosettes were isolated using STEMdiff Neural Rosette Selection Reagent and replated on poly-L-ornithine/laminin-coated plates for further NPCs expansion. The NPCs were either banked or used directly for differentiation and expanded up to 4 passages.

For differentiation into neural precursors, NPCs were cultured for 7 days in STEMdiff Neural Progenitor Medium, then dissociated with accutase and plated in STEMdiff Forebrain Neuron Differentiation Medium for an additional week. Neural precursors were matured using the STEMdiff Forebrain Neuron Maturation Kit. All culture steps were performed on tissue culture-treated plastic plates, with 24-well glass-bottom plates used for imaging and immunocytochemical analyses. This differentiation protocol reliably produces validated NPCs, precursor cells, and 10 weeks old forebrain neurons suitable for downstream analyses.

### IMMUNOCYTOCHEMISTRY

For immunocytochemistry (ICC) on cultured cells, culture media was removed, and cells were washed twice with 1X PBS. The cells were then fixed in 4% paraformaldehyde prepared in 1X PBS for 15 minutes at room temperature (RT). Following fixation, cells were washed twice with 1X PBS and blocked in blocking solution consisting of 1X PBS with 0.3 M glycine, 0.2% Triton X-100, and 5% normal donkey serum for 60 minutes at RT.

For 5-ethynyl-2’-deoxyuridine **(EdU)** detection, cells were processed prior to the blocking step following the manufacturer’s instructions provided in the Click-iT EdU Cell Proliferation Kit (Invitrogen). Primary antibodies were diluted in blocking solution and cells were incubated for overnight at 4°C. Antibodies used in experiments are listed in Table S1. After primary antibody incubation, cells were washed three times with TBST (PBS with 0.1% Tween-20) for 5 minutes each and incubated with fluorescently labeled secondary antibodies diluted in TBST solution for 2h at RT. Cells were then washed three times with TBST, counterstained with DAPI, and mounted using ProLong Gold Antifade Mountant (Invitrogen). Images were acquired at 20x magnification using a Keyence BZ-X800 microscope.

### WESTERN BLOTTING

Cells were washed three times with ice-cold 1X PBS and lysed on ice for 10 minutes using RIPA buffer supplemented with protease and phosphatase inhibitor cocktails (Thermo Fisher Scientific). Lysates were sonicated on ice at 20% power for three cycles of 15 seconds each, with 5-second rests between sonication. Following sonication, samples were centrifuged at 10,000 x g for 15 minutes at 4°C to pellet insoluble debris, and the clarified supernatant was collected. Protein concentrations were determined using the Pierce BCA Protein Assay Kit (Thermo Fisher Scientific).

For electrophoresis, equal amounts of protein were mixed with NuPAGE LDS Sample Buffer and NuPAGE Reducing Agent (Invitrogen), then denatured by boiling at 95°C for 5 minutes. Samples were resolved on Bolt Bis-Tris Plus SDS-PAGE gels (Thermo Fisher Scientific) using Bolt MES SDS Running Buffer. Proteins were transferred to nitrocellulose membranes using the iBlot 3 Dry Blotting System (Invitrogen). Membranes were blocked for 1 hour at room temperature in 5% non-fat dry milk prepared in Tris-buffered saline with 0.1% Tween-20 (TBST). Primary antibodies diluted in 5% milk/TBST were incubated with membranes overnight at 4°C. Antibodies used in experiments are listed in Table S1. After washing membranes three times for 15 minutes each in TBST, membranes were incubated with horseradish peroxidase-conjugated secondary antibodies diluted in 5% milk/TBST for 2 hours at room temperature. Following secondary antibody incubation, membranes were washed three times as before and developed using SuperSignal West Pico PLUS Chemiluminescent Substrate (Thermo Fisher Scientific). Blots were imaged with the Azure Biosystems 300q Image Western Blot Imaging System.

Densitometric quantification of band intensities was performed using Fiji. Protein expression levels were normalized to housekeeping controls such as GAPDH.

### ENZYME-LINKED IMMUNOSORBENT ASSAY FOR AMYLOID-BETA IN NEURONAL CONDITIONED MEDIA

Conditioned media were collected from 10 weeks old neuronal cultures derived from iPSCs. For conditioned media samples, cells were rinsed three times with Dulbecco’s phosphate-buffered saline (DPBS) to remove residual media, then cultured in 1 mL of fresh media for 48 hours. Following incubation, conditioned media were harvested and snap-frozen in liquid nitrogen and stored until analysis. To quantify amyloid-beta isoforms, Human β Amyloid (1-40) Enzyme-Linked Immunosorbent Assay (ELISA) Kit Wako II (Fujifilm Irving, 298-64601) and Human β Amyloid (1-42) ELISA Kit Wako (Fujifilm Irving, 298-62401) were used according to the manufacturer’s protocols. Conditioned media samples were diluted 1:4 for Aβ40 measurement and 1:2 for Aβ42 measurement before loading. All ELISA plates were read on an microplate reader at the 450nm wavelength, and data were processed following kit guidelines. Resulting amyloid-beta concentrations were normalized to the total protein extracted from the corresponding wells.

### STATISTICS

In all graphs, data is shown as mean ± SEM. Prism (Graphpad) was used for statistical analysis with tests indicated in figure legends. The following was used for p values: *p < 0.05, **p < 0.01, ***p < 0.001, and ****p < 0.0001.

## Results

### DOWN SYNDROME INDUCED PLURIPOTENT STEM CELLS-DERIVED NEURAL CELLS EXHIBIT REDUCED PROLIFERATION AND PREMATURE NEURONAL DIFFERENTIATION.

We first examined the course of neuronal differentiation using DS patient- derived iPSC (DS) and isogenic (Ctrl) lines (Figure 1A). The iPSC lines were differentiated into NPCs followed by neural precursor cells and forebrain neurons using neural induction method (Figure 1B). We assessed **c**ell proliferation of NPCs using Click-It EdU assay. We observed a decrease in the proliferative capacity of DS NPCs compared to Ctrl, as indicated by a significantly reduced percentage of EdU+ cells in DS NPCs (Figure 1C–D). Further, the trajectory of neuronal differentiation progression was assessed by quantifying the expression of SOX2, DCX, and βIII-Tubulin in the cultures. As expected, immunofluorescence studies assessing SOX2 expression in NPCs, precursor and forebrain neurons revealed a decrease in SOX2+ cells across differentiation stages in both DS and Ctrl. However, DS lines showed a moderate but significant increase in SOX2+ cells compared to Ctrl at each differentiation stage. (Figure 1E–F). In addition, a significant increase in the number of DCX+ cells was observed at each stage of neurogenesis in DS compared to Ctrl (Figure 1G–H). **Lastly,** a significant increase in the number of β-III-tubulin+ neurons was observed in the DS lines compared to Ctrl (Figure 1I–J).

**Figure 2.**
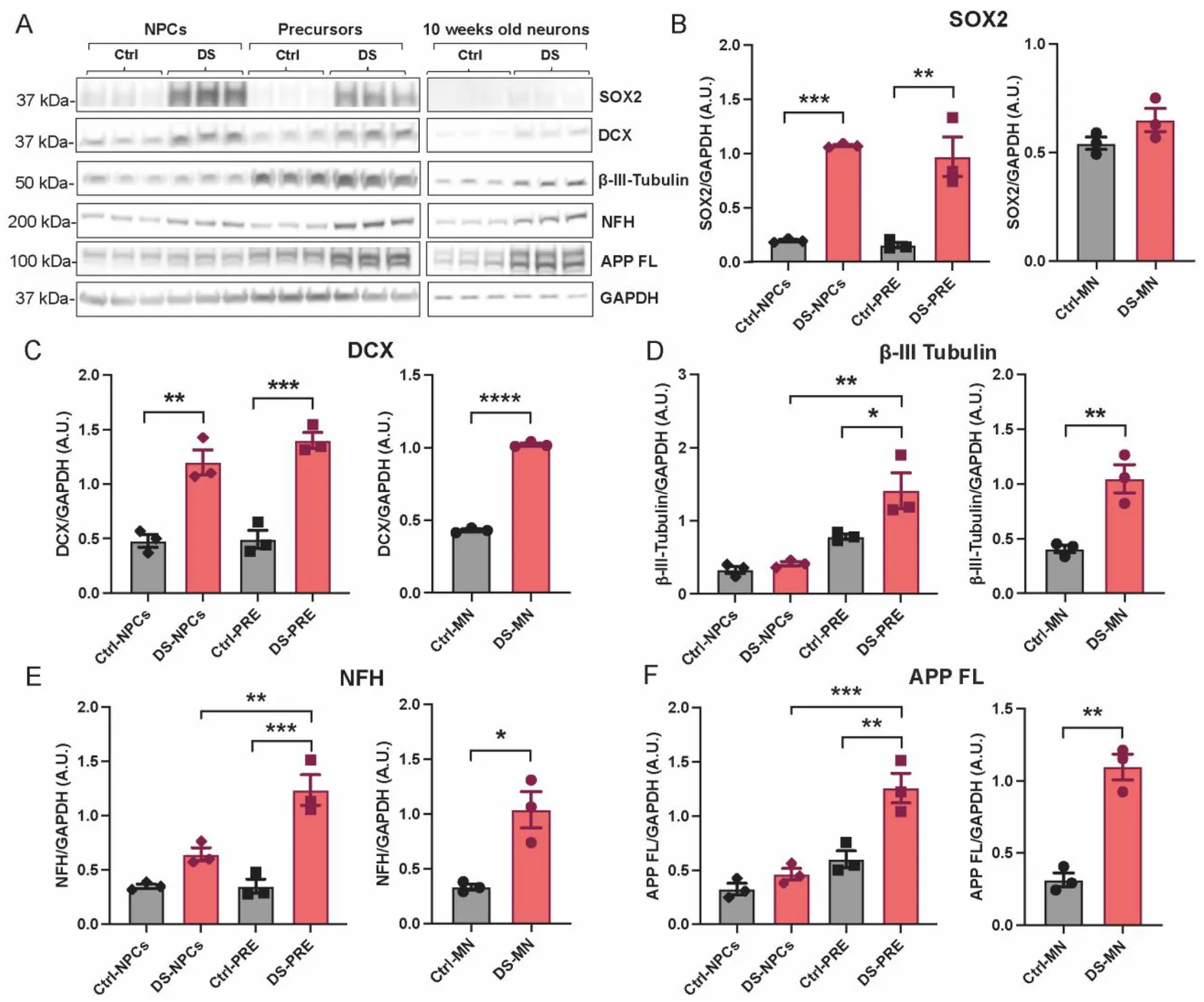
Altered protein expression profile of key neurogenesis signals in Down Syndrome induced pluripotent stem cells-derived neural populations. (A) Expression levels of SOX2, DCX (doublecortin), β-III-Tubulin, neurofilament heavy chain (NFH), and APP full-length (APP FL) in NPCs, PRE, and 10-week-old neurons (MN) derived from Control (Ctrl) and Down Syndrome (DS) iPSCs, as detected by Western blot analysis. (B, C, D, E, F) Quantification of SOX2, DCX, β-III-Tubulin, NFH, and APP FL expression normalized to GAPDH in NPCs, precursor cells (left) and neurons differentiated from control and DS groups (right) supports increased neuronal differentiation in the DS group (n=3 replicates). Data represented as mean ± SEM with individual data points shown. Data analyzed by unpaired t test (neurons), one-way ANOVA with Tukey’s multiple comparisons test (NPCs and precursor cells data), *p < 0.05, **p < 0.01, ***p < 0.001, and ****p < 0.0001.

To further validate the neuronal differentiation phenotypes, we examined the expression levels of these proxies by Western blot analysis (Figure 2A). Results revealed significant alterations in the expression of neuronal proxies during the differentiation of DS iPSCs-derived neural cells compared to Ctrl. Specifically, and in agreement with the immunofluorescence analysis, we observed increased expression of SOX2 in NPCs and precursors in the DS lines compared to Ctrl (Figure 2A,B). In addition, we observed increased DCX (Figure 2A,C) and β-III-tubulin (Figure 2A,D) expression in NPCs, precursors and 10-week-old neurons in the DS lines. Finally, there was an increase in neurofilament heavy chain in the 10-week-old DS neurons (Figure 2A,E). Together, this is indicative of disruptive neurogenesis that includes prolonged stemness along with enhanced neuronal maturation. Collectively, these results indicate that DS iPSCs-derived neural cells display reduced proliferative output with altered neurogenic state progression reflected by persistently increased SOX2+ cells and elevated neuronal lineage markers, mirroring neurodevelopmental abnormalities observed in Down Syndrome^27,28^.

### ENHANCED AMYLOIDOGENIC AMYLOID PRECURSOR PROTEIN PROCESSING AND TAU ACCUMULATION IN DOWN SYNDROME INDUCED PLURIPOTENT STEM CELLS-DERIVED NEURONS.

**Figure 3.**
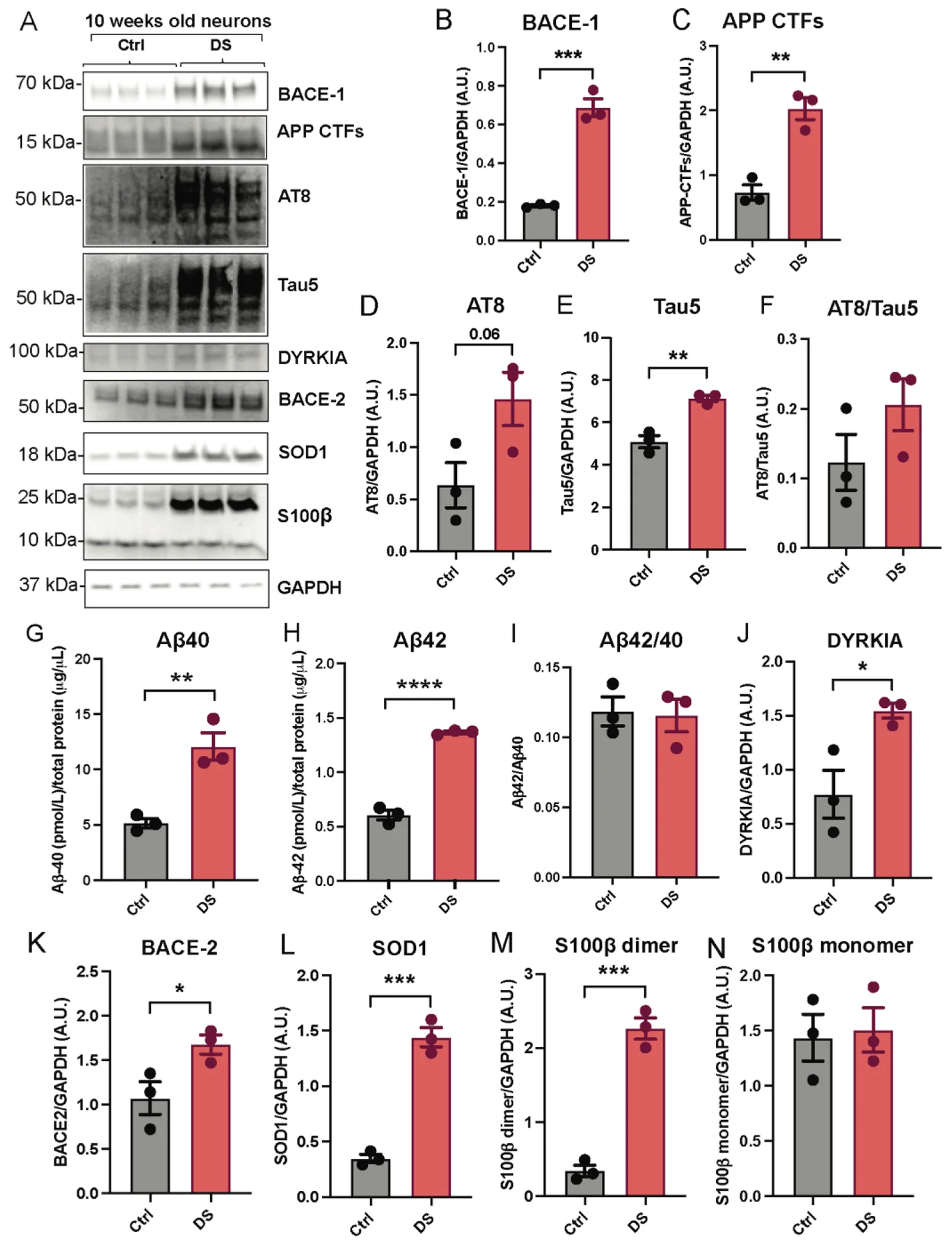
Increased amyloidogenic processing of amyloid precursor protein, tau, and upregulation of chromosome 21 genes and stress response proteins in Down Syndrome induced pluripotent stem cells-derived neurons. (A) Increased expression of BACE-1, APP C-terminal fragments (CTFs), phosphorylated tau (AT8), total tau (Tau5), DYRK1A, BACE-2, SOD1, and S100β in protein lysate of 10-week-old neurons derived from DS iPSCs compared to Ctrl, as detected by Western blot analysis. (B-E, J-N) Quantification of BACE-1, total APP CTFs, AT8, Tau5, DYRK1A, BACE-2, SOD1, and S100β levels normalized to GAPDH in Ctrl and DS groups. (n = 3 replicates). (F) AT8/Tau5 ratio indicates no significant difference between DS and Ctrl groups. (G-H) Quantification of secreted Aβ_40_ and Aβ_42_ in DS neuronal cultures compared to controls using ELISA. (n = 3 replicates). (I) The Aβ_42_/Aβ_40_ ratio is comparable between genotypes. Data represented as mean ± SEM with individual data points shown. Data analysed by unpaired t test, *p < 0.05, **p < 0.01, ***p < 0.001, and ****p < 0.0001.

To start to address the role of APP in this developmental trajectory, we first assessed the protein levels of FL-APP (full length APP) at differentiation stages of cortical neurogenesis. Gene dosage effect of HSA21 triplication was apparent at the precursor stage; FL-APP was upregulated in DS precursors and neurons compared to Ctrl counterparts (Figure 2A, F). Examination of FAD-linked signalling revealed elevated levels of BACE-1 (Figure 3B), which was associated with significantly higher levels of APP C-terminal fragments (APP CTFs, Figure 3C), suggesting enhanced BACE-1 activity in DS relative to Ctrl. Down syndrome neurons also exhibited increased total tau levels (Figure 3E), with a trend toward higher phosphorylated tau (AT8, Figure 3D). The ratio of phosphorylated to total tau (AT8/Tau5) did not differ significantly (Figure 3F), suggesting that the increase in p-tau is due to increased tau, may be due to enhanced neuronal differentiation in DS, rather than AD pathology. A marked increase in secretion of both Aβ_40_ (Figure 3G) and Aβ_42_ (Figure 3H) peptides was observed in conditioned media of 10-weeks old DS neurons. Notably, the Aβ_42_/Aβ_40_ ratio remained unchanged between groups, supporting an increase in Aβ due to enhanced BACE-1 activity (Figure 3I). Together, these results suggest that trisomy 21 drives a coordinated shift toward amyloidogenic APP processing in human DS neurons, even at an early maturation stage.

### UPREGULATION OF CHROMOSOME 21 ENCODED PROTEINS IN DOWN SYNDROME NEURONS.

Next, we asked whether alterations in expression of additional genes on HSA21 would be apparent in our iPSC-derived DS neurons. Particularly, selected genes that are implicated in inflammation, neurodegeneration and neurogenesis, DYRK1A, BACE2, SOD1 and S100β. Quantitative analysis of protein expression in 10-week-old iPSCs-derived neurons revealed the upregulation of DYRK1A (Figure 3J), BACE-2 (Figure 3K) **and** SOD1 (Figure 3L) **in the DS neurons compared to Ctrl.** Notably, DS neurons showed a robust increase in S100β dimer levels (Figure 3M), while monomer levels remained unchanged between groups (Figure 3N). This suggests a shift in the oligomeric state of S100β in DS neurons and may indicate altered S100β signaling or stress responses without a change in total S100β production^30,31^. Oligomeric S100β appeared as a prominent dimer band, consistent with formation of disulfide-linked S100β dimers that are preserved in the mildly reducing, oxidizing environment of DS neurons^32–34^. Together, these findings highlight the pervasive impact of chromosome 21 trisomy on gene and protein expression profiles, implicating these molecular changes in disrupted cellular homeostasis and heightened stress responses in DS-derived neural populations.

### NORMALIZATION OF *AMYLOID PRECURSOR PROTEIN* COPY NUMBER RESCUES NEUROGENESIS DEFICITS IN DOWN SYNDROME INDUCED PLURIPOTENT STEM CELLS-DERIVED NEURONS.

**Figure 4.**
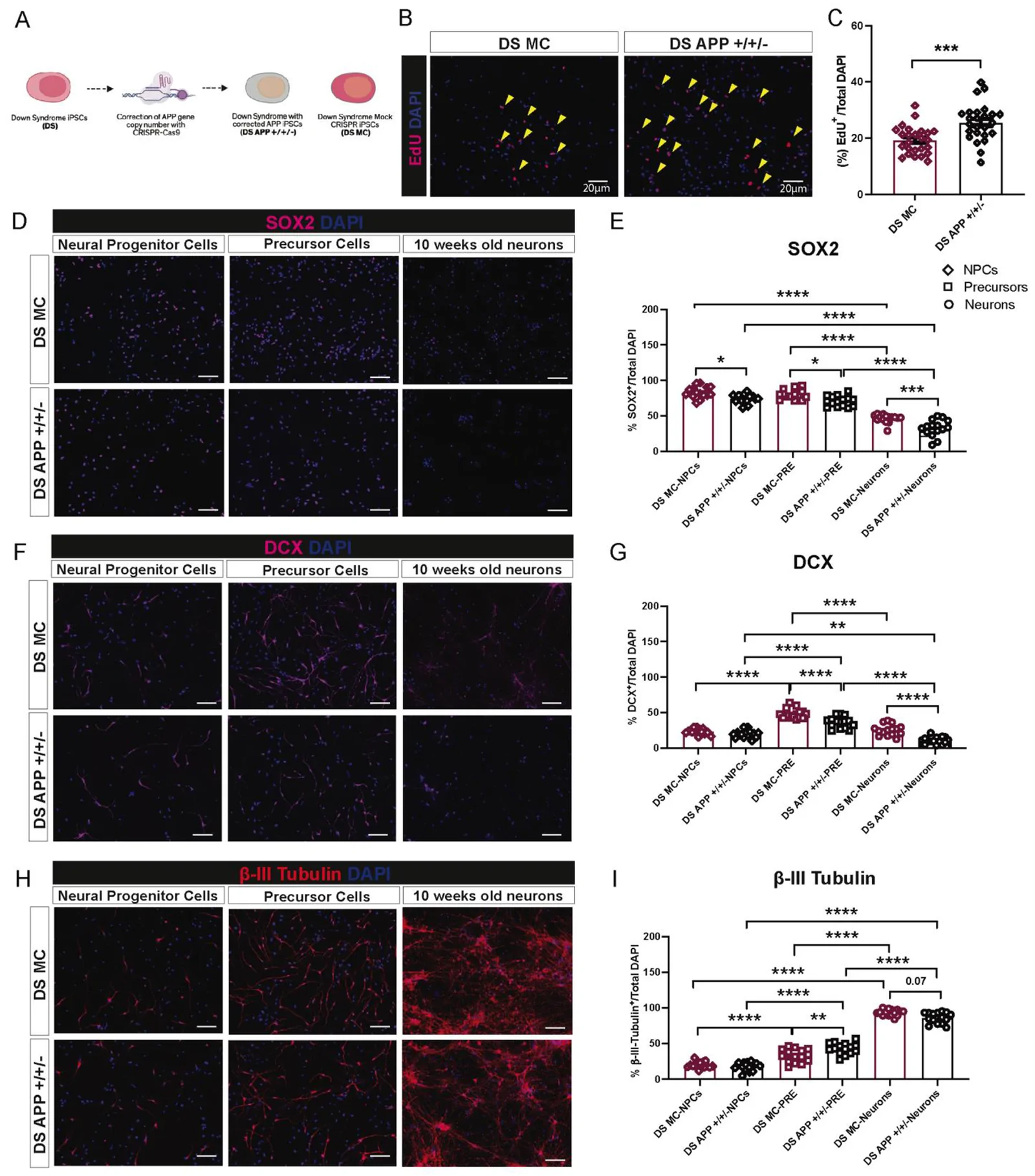
Correction of amyloid precursor protein gene dosage rescues proliferation and neuronal differentiation defects in Down Syndrome induced pluripotent stem cells-derived cells. (A) Schematic depicting the manipulation of DS iPSCs with either mock CRISPR editing (DS MC) or APP gene copy correction (DS APP+/+/−). (B) Representative immunofluorescence images showing EdU incorporation (red) and DAPI (blue) in NPCs from DS MC and DS APP+/+/− lines. Yellow arrowheads indicate EdU+ proliferating cells. Scale bar: 20μm. (C) Quantification of EdU+ cells as a percentage of total DAPI-labelled nuclei, suggesting significantly increased proliferation in DSAPP+/+/− NPCs compared to DS MC. (D, F, H) Representative images of SOX2 (magenta), DCX (magenta), β-III-Tubulin (red), and DAPI (blue) immunostaining in NPCs, precursor cells, and 10 weeks old neurons derived from DS MC and DS APP+/+/− iPSCs. Scale bars: 20μm. (E, G, I) Quantification of SOX2+, DCX+, and β-III-Tubulin+ cells (% of total DAPI) across differentiation stages. n=9 images per group. Data represented as mean ± SEM with individual points shown. Data analyzed by unpaired t-test (C), two-way ANOVA with Sidak’s multiple comparisons test (E, G, I), *p < 0.05, **p < 0.01, ***p < 0.001, and ****p < 0.0001.

Having established the phenotypes of DS iPSC-derived neurogenesis and neurons, we examined the hypothesis that increased APP gene dosage contributes to deficits in neurogenesis and the development of AD pathology in DS. For that, we used a DS patient-derived iPSCs in which one APP allele was silenced by CRISPR/Cas9 (DS APP *+/+/−*), and a mock CRISPR-Cas9 DS control (DS MC) (Figure 4A). Driven by this hypothesis, we investigated whether the restoration of *APP* gene dosage in DS iPSCs would reverse neurogenesis deficits. Our results showed that DS APP*+/+/−* cells exhibited a marked increase in the percentage of proliferating NPCs, as shown by greater EdU incorporation compared to DS MC (Figure 4B–C). Immunofluorescence quantification further demonstrated restoration of SOX2+ cell number at all three stages of differentiation (Figure 4D–E) and normalization of the number of DCX+ cells at neuronal precursor and neuronal stages in DS APP*+/+/−* group (Figure 4F–G). Moreover, the number of β-III-tubulin+ neurons was normalized in DS APP*+/+/−* compared to DS MC (Figure 4H–I).

**Figure 5.**
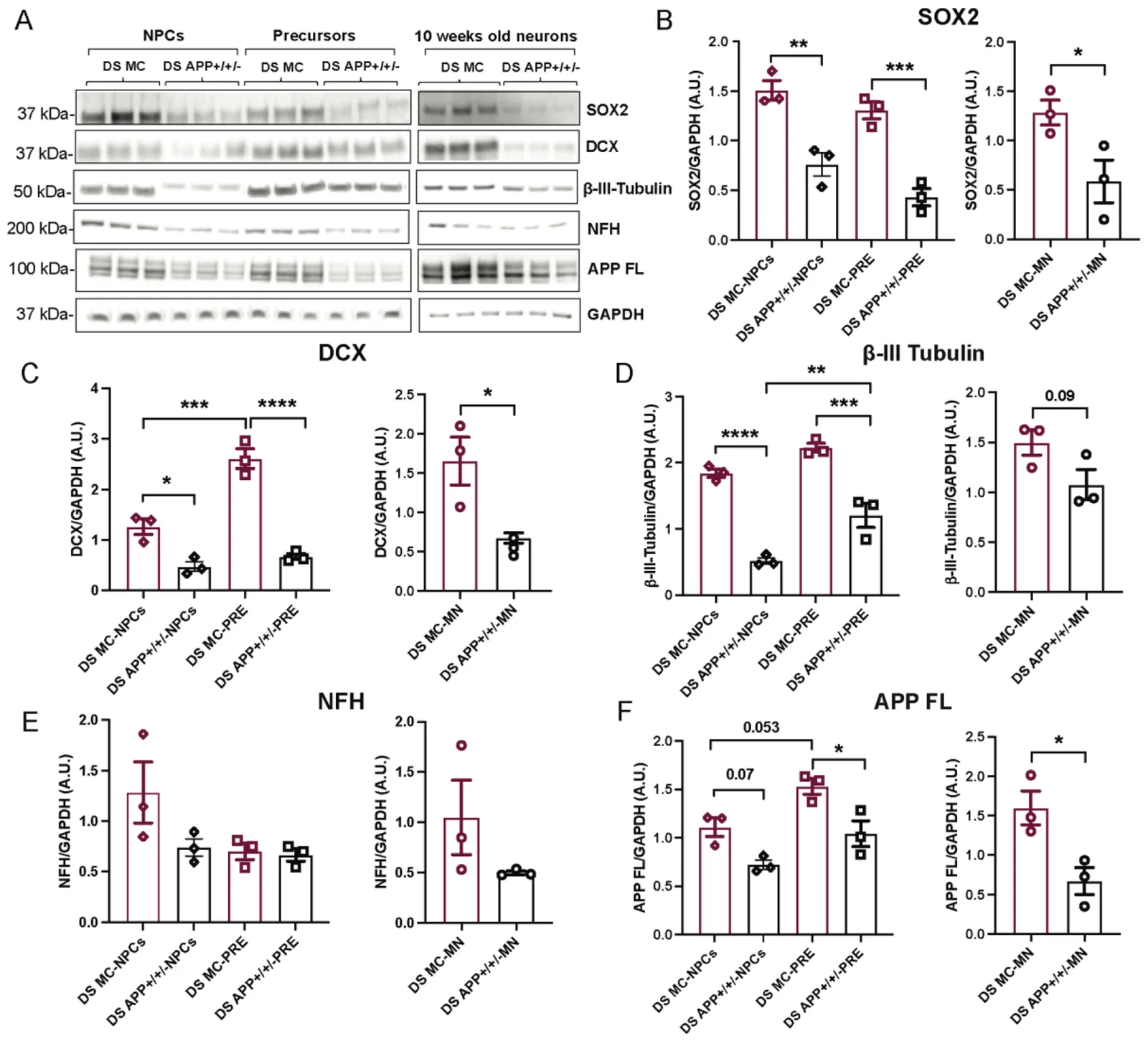
Amyloid precursor protein dosage correction reverses aberrant neurogenesis proxy expression in Down Syndrome induced pluripotent stem cells-derived cells. (A) Expression levels of SOX2, DCX (doublecortin), β-III-Tubulin, neurofilament heavy chain (NFH), and APP full-length (APP FL) across NPCs, precursor cells, and 10 week-old-neurons (MN) derived from DS iPSCs with DS MC and DS APP+/+/− groups, as detected by Western blot. (B, C, D, E, F) Quantification of SOX2, DCX, β-III-Tubulin, NFH, and APP FL expression normalized to GAPDH in NPCs and precursor stages (left) and in neurons differentiated from APP protein levels in DS APP+/+/− and DS MC iPSC lines, (n = 3 replicates). Data represented as mean ± SEM with individual data points shown. Data analyzed by unpaired t- test (neurons), one-way ANOVA with Tukey’s multiple comparisons test (NPCs and precursor cells data), *p < 0.05, **p < 0.01, ***p < 0.001, and ****p < 0.0001.

To further validate these findings, we examined the expression levels of these proxies in protein lysates of these lines. Normalization of *APP* gene dosage in DS iPSCs-derived neural cultures validated the robust rescue of aberrant neurogenesis proxies (Figure 5A). The DS APP*+/+/−* NPCs, precursors and neurons exhibited markedly reduced levels of SOX2 (Figure 5A, B), DCX (Figure 5A, C), and β-III-tubulin (Figure 5A, D) compared to DS MC controls. Western blot analysis demonstrated no significant change in NFH protein levels in both the groups (Figure 5A, E). Collectively, these results indicate that restoring *APP* gene dosage corrects the detected proliferative phenotype and to some extent differentiation abnormalities in DS iPSCs-derived neural stem cells.

### RESCUE OF ALZHEIMER’S DISEASE MOLECULAR PHENOTYPES BY AMYLOID PRECURSOR PROTEIN DOSAGE NORMALIZATION IN DOWN SYNDROME NEURONS.

**Figure 6.**
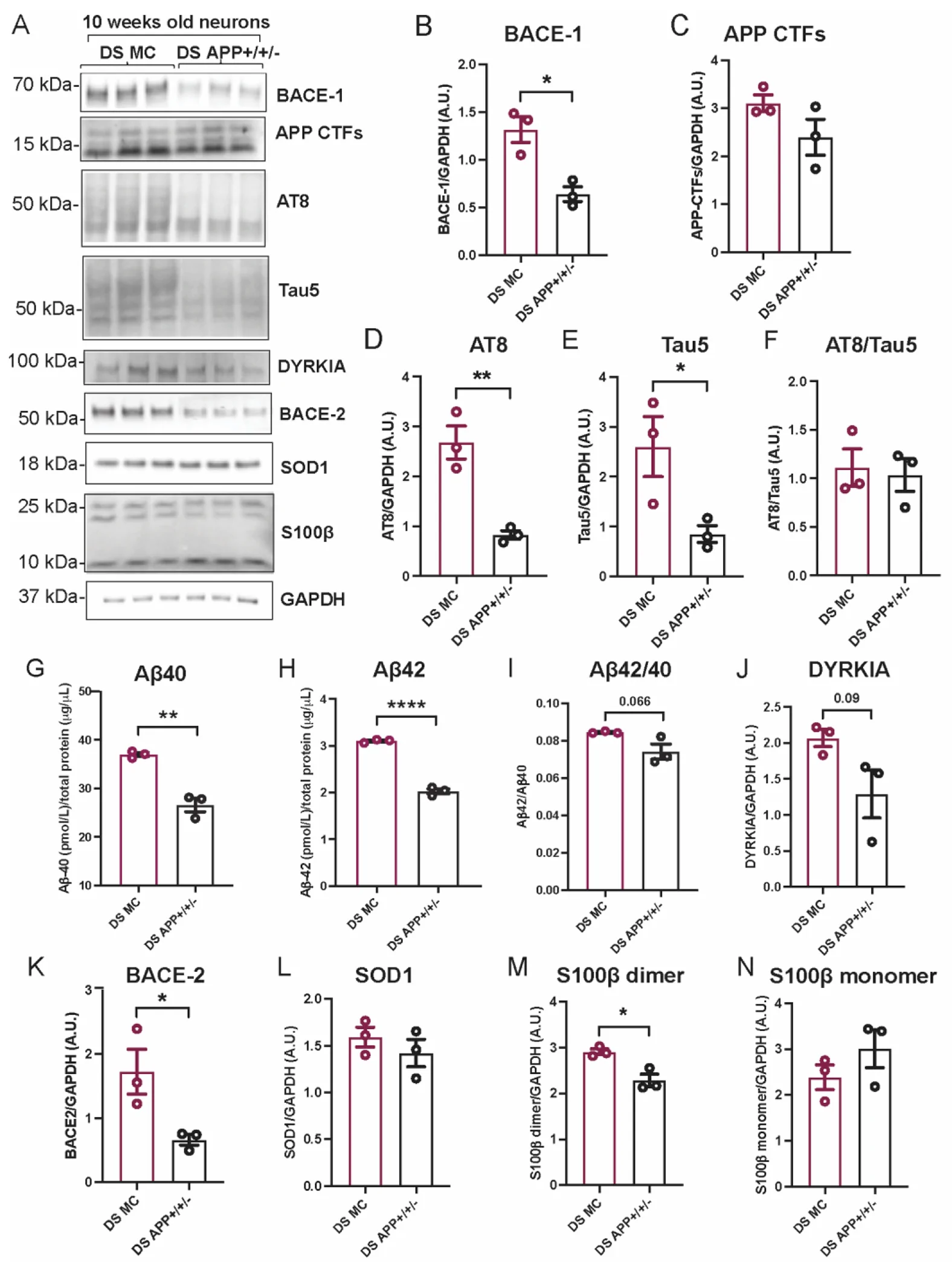
Amyloid precursor protein dosage correction attenuates Alzheimer’s disease related pathology and chromosome 21 gene and stress response protein dysregulation in Down Syndrome induced pluripotent stem cells-derived neurons. (A) Expression levels of BACE-1, APP C-terminal fragments (APP CTFs), phosphorylated tau (AT8), total tau (Tau5), DYRK1A, BACE-2, SOD1, and S100β in protein lysate of 10 week old neurons (MN) derived from DS MC and DS APP+/+/− iPSC lines, as examined by Western blot. (B-E, J-N) Quantification of BACE-1, APP CTFs, AT8, Tau5, DYRK1A, BACE-2, SOD1, and S100β levels normalized to GAPDH in DS MC and DS APP+/+/− groups. (n=3 replicates). (F) The ratio of phosphorylated- to total- tau (AT8/Tau5) is comparable between genotypes. (G, H) Quantification of secreted Aβ_40_ and Aβ_42_ in conditioned media of DS APP^+/+/−^ vs DS MC neuronal cultures using ELISA. (n=3 replicates). (I) Aβ_42_/Aβ_40_ ratio following APP dosage correction. Data represented as mean ± SEM with individual data points shown. Data analyzed by unpaired t-test, *p < 0.05, **p < 0.01, and ****p < 0.0001.

Next, we asked whether the normalization of *APP* copy number and dose, rescues the increase in APP processing and subsequent AD-linked pathology. We observed that FL-APP protein levels were reduced at all stages in DS APP*+/+/−* lines compared to DS **MC** (Figure 5A, F), confirming successful genetic correction. Furthermore, normalization of *APP* gene dosage in DS iPSCs-derived neurons profoundly attenuates alterations in AD-related signals. Specifically, we observed significantly reduced levels of BACE-1 in DS APP*+/+/−* neurons compared to DS MC (Figure 6A–B). **Levels** of APP-CTFs were comparable between the genotypes (Figure 6A, C). Both AT8 (Figure 6A, D) and Tau5 (Figure 6A, E) were markedly diminished in DS APP*+/+/−* neurons compared to DS MC, suggesting normalization of tau metabolism. The ratio of AT8/Tau5 remained unchanged (Figure 6F), suggesting that *APP* correction primarily reduces overall tau levels rather than alters phosphorylation stoichiometry. Levels of secreted Aβ_40_ (Figure 6G) and Aβ_42_ (Figure 6H) were robustly decreased in DS APP*+/+/−* neurons. Aβ_42_/Aβ_40_ ratio showed trend, albeit not statistically significant (Figure 6I). Collectively, these results demonstrate that restoring *APP* dosage levels in DS iPSCs-derived neurons rescues key AD-associated biochemical phenotypes.

### *AMYLOID PRECURSOR PROTEIN* GENE NORMALIZATION DIMINISHES CHROMOSOME 21 GENE DOSAGE EFFECTS AND STRESS PATHWAYS IN DOWN SYNDROME NEURONS.

Finally, we examined whether the normalization of *APP* gene dosage in DS iPSCs-derived neurons had an effect on HSA21 genes that were observed to be upregulated (Figure 3). Examination of protein lysate of DS APP*+/+/−* and DS MC neurons revealed no significant change in DYRK1A, but significantly lower levels of BACE2 in DS APP*+/+/−* neurons compared to DS MC (Figure 6A, J–K). Levels of SOD1 did not change (Figure 6A, L). Notably, S100β dimer and monomer levels were comparable in both genotypes (Figure 6A, M,N). These data indicate that *APP* gene dosage in DS neurons partially affects the dysregulation of genes on HSA21, underscoring the central regulatory role of APP in DS neuropathology.

## Discussion

This study reveals several novel aspects. First, we show that APP overdose impairs DS NPC proliferation. Interestingly, we observed upregulation of SOX2 expression in NPCs and precursor cells and increased number of cells expressing SOX2. Together, this may suggest reduced pools of progenitor cells with enhanced stemness due to APP overdose. In turn, APP overdose induced premature differentiation, based on the expression of neurogenesis proxies in DS forebrain neurons. This may suggest that APP regulates more than one checkpoint along the neurogenic path. This central role of APP in DS brain development aligns with previous reports showing increased APP expression in DS fetal brain, leading to impaired neural progenitor proliferation and a gliocentric shift^34,35^. Recent evidence demonstrates that APP is broadly expressed in human cortical progenitors throughout neurogenesis and is required for maintaining the temporal balance between self-renewal and differentiation of human neural stem cells. Loss of APP in human neural progenitors drives premature cell cycle exit, and increased generation of human cortical neurons. Importantly, APP modulates neurogenesis through proneurogenic activator protein-1 transcription factor suppression and activation of WNT signaling^36^. Notably, our data suggests that HSA21 trisomy enhances BACE-1 expression and activity, leading to biased increase in APP metabolites cleaved by BACE-1. Future studies are warranted to determine the effect of these metabolites on these impairments. Studies have found elevated APP and its cleavage products, higher levels of Aβ, phospho-tau, and corresponding neurodevelopmental and neurodegenerative alterations using DS iPSC-derived neurons^35,37^. Experimental reduction in APP and its products using Posiphen, has been shown to partially restore AD-linked neuronal phenotypes in mouse models^37,38^. Notably, evidence suggests that metabolites of APP, which is mutated in familial forms of AD, play critical roles in developmental and postnatal neurogenesis. Of particular importance is soluble APP (sAPP), which regulates neural progenitor cell proliferation^15^. This highlights the essential roles of APP metabolites in neurogenesis, NPC proliferation, and fate specification. In addition, future studies should examine the implications for neuronal function. In that regard, we observed upregulation of tau species in DS neurons. Given the importance of tau in neuronal development, its upregulation may compromise proper neuronal maturation. Notably, we did not observe increased tau phosphorylation relative to total tau, suggesting that other factors may play a role in tau hyperphosphorylation or that AD-linked hyperphosphorylation takes place at later stages of brain function. One possibility is that a two-hit, such as aging and APP overdose, would be required for the pathological acceleration of tau hyperphosphorylation. While we observed enhanced expression of BACE-1 and enhanced secretion of Aβ_42_ and Aβ_40_ in DS neurons, in contrast to previous studies^11^, we did not observe altered Aβ_42_/Aβ_40_ ratio. Possible explanations to this unaltered Aβ_42_/Aβ_40_ ratio could be due to differences in differentiation protocols, maturation state, or APP processing enzyme expression, as well as the possibility that APP dosage modulation in this early developmental window predominantly affects amyloidogenic output rather than selectively shifting the Aβ_42_/Aβ_40_ balance. Future work should examine long-term cultures and manipulations of specific secretases that may clarify how APP dosage affects its processing by γ-secretase.

Besides APP, our data suggests that several other genes on chromosome 21 contribute to the complex neuropathology of DS. DYRK1A, a kinase implicated in neurodevelopmental processes, is consistently upregulated in DS neurons and has been shown to influence neuronal proliferation, differentiation, and tau phosphorylation, thereby linking it to both developmental abnormalities and Alzheimer-like pathology^39,40^. The BACE2 is also overexpressed, although its exact role in DS remains to be fully elucidated. Additionally, the calcium-binding protein S100 shows differential regulation in its dimeric and monomeric forms; notably, increased S100 dimer levels in DS neurons indicate heightened stress and inflammatory responses, whereas monomeric levels remain unchanged^41^. Notably, our data shows that elevated protein levels of BACE-2 in DS iPSC-derived neurons were reversed by normalization of APP gene dosage, suggesting that BACE-2 dysregulation is dependent or downstream of APP-mediated pathways. However, SOD1, DYRKIA, and S100β, were not rescued by APP gene dosage normalization, indicating that their dysregulation may be independent- or upstream- of APP pathways. Evidence from the literature supports that SOD1 overexpression exacerbates oxidative damage and impacts neuronal differentiation and survival^42,43^, implicating oxidative stress as a complementary mechanism alongside APP overdose in the pathogenesis of DS neurodevelopmental and neurodegenerative phenotypes^44^. Together, these gene dosage effects underscore the multifaceted molecular disruptions driving neurodevelopmental and neurodegenerative phenotypes in DS. Future studies should focus on dissecting the specific mechanistic contributions of these genes to better understand this disorder.

Limitations of our study include the 2D differentiation which does not allow full assessment of corticogenesis, and the lack of mixed cellular interactions with glia and microglia. The data is obtained from 10-week- old neurons, a relatively short culture duration, which does not allow aging-related processes, as well as the accumulation of amyloid deposition and neurofibrillary tangles. Future studies should use these iPSC lines for the development of cortical organoids with prolonged maturation to model more advanced DS-AD pathology. The DS MC and DS DS APP*+/+/−* lines provide a platform for the examination of the effect of APP overdose on many aspects of developmental neurogenesis and AD pathology that are yet to be elucidated.

## Conclusion

In summary, this study suggests that APP overdose causes impairments in neurogenesis and facilitates amyloidosis in DS. Integrating these insights builds a nuanced view of APP’s role or its downstream pathways, in DS, as a strategy to ameliorate both neurodevelopmental and neurodegenerative sequelae in DS.

## Supplementary Material

1

## References

[1] Ruparelia A, Pearn ML, Mobley WC. Aging and intellectual disability: insights from mouse models of Down syndrome. Dev Disabil Res Rev. 2013;18(1):43–50. doi:10.1002/ddrr.1127 23949828

[2] Patkee PA, Baburamani AA, Kyriakopoulou V, Early alterations in cortical and cerebellar regional brain growth in Down Syndrome: An in vivo fetal and neonatal MRI assessment. NeuroImage Clin. 2020;25:102139. doi:10.1016/j.nicl.2019.102139 31887718 PMC6938981

[3] Dierssen M. Down syndrome: the brain in trisomic mode. Nat Rev Neurosci. 2012;13(12):844–858. doi:10.1038/nrn3314 23165261

[4] Korbel JO, Tirosh-Wagner T, Urban AE, The genetic architecture of Down syndrome phenotypes revealed by high-resolution analysis of human segmental trisomies. Proc Natl Acad Sci U S A. 2009;106(29):12031–12036. doi:10.1073/pnas.0813248106 19597142 PMC2709665

[5] Müller UC, Deller T, Korte M. Not just amyloid: physiological functions of the amyloid precursor protein family. Nat Rev Neurosci. 2017;18(5):281–298. doi:10.1038/nrn.2017.29 28360418

[6] Young-Pearse TL, Bai J, Chang R, Zheng JB, LoTurco JJ, Selkoe DJ. A critical function for beta-amyloid precursor protein in neuronal migration revealed by in utero RNA interference. J Neurosci Off J Soc Neurosci. 2007;27(52):14459–14469. doi:10.1523/JNEUROSCI.4701-07.2007 PMC6673432 18160654

[7] Coronel R, López-Alonso V, Gallego MI, Liste I. Low Levels of Amyloid Precursor Protein (APP) Promote Neurogenesis and Decrease Gliogenesis in Human Neural Stem Cells. Int J Mol Sci. 2023;24(19):14635. doi:10.3390/ijms241914635 37834082 PMC10572469

[8] Caillé I, Allinquant B, Dupont E, Soluble form of amyloid precursor protein regulates proliferation of progenitors in the adult subventricular zone. Dev Camb Engl. 2004;131(9):2173–2181. doi:10.1242/dev.01103 15073156

[9] Rice HC, Townsend M, Bai J, Pancortins interact with amyloid precursor protein and modulate cortical cell migration. Dev Camb Engl. 2012;139(21):3986–3996. doi:10.1242/dev.082909 PMC3472593 22992957

[10] Müller UC, Deller T, Korte M. Not just amyloid: physiological functions of the amyloid precursor protein family. Nat Rev Neurosci. 2017;18(5):281–298. doi:10.1038/nrn.2017.29 28360418

[11] Lee SH, Kang J, Ho A, Watanabe H, Bolshakov VY, Shen J. APP Family Regulates Neuronal Excitability and Synaptic Plasticity but Not Neuronal Survival. Neuron. 2020;108(4):676–690.e8. doi:10.1016/j.neuron.2020.08.011 32891188 PMC7704911

[12] Klevanski M, Herrmann U, Weyer SW, The APP Intracellular Domain Is Required for Normal Synaptic Morphology, Synaptic Plasticity, and Hippocampus-Dependent Behavior. J Neurosci. 2015;35(49):16018–16033. doi:10.1523/JNEUROSCI.2009-15.2015 26658856 PMC6605503

[13] Hick M, Herrmann U, Weyer SW, Acute function of secreted amyloid precursor protein fragment APPsα in synaptic plasticity. Acta Neuropathol (Berl). 2015;129(1):21–37. doi:10.1007/s00401-014-1368-x 25432317

[14] Li H, Wang Z, Wang B, Genetic dissection of the amyloid precursor protein in developmental function and amyloid pathogenesis. J Biol Chem. 2010;285(40):30598–30605. doi:10.1074/jbc.M110.137729 20693289 PMC2945554

[15] Lazarov O, Demars MP. All in the Family: How the APPs Regulate Neurogenesis. Front Neurosci. 2012;6:81. doi:10.3389/fnins.2012.00081 22675290 PMC3366480

[16] Zhou Z dong, Chan CH shan, Ma Q hong, Xu X hong, Xiao Z cheng, Tan EK. The roles of amyloid precursor protein (APP) in neurogenesis, implications to pathogenesis and therapy of Alzheimer disease (AD). Cell Adhes Migr. 2011;5(4):280–292. doi:10.4161/cam.5.4.16986 PMC3210295 21785276

[17] Wang B, Wang Z, Sun L, The Amyloid Precursor Protein Controls Adult Hippocampal Neurogenesis through GABAergic Interneurons. J Neurosci. 2014;34(40):13314–13325. doi:10.1523/JNEUROSCI.2848-14.2014 25274811 PMC4180470

[18] Demars MP, Bartholomew A, Strakova Z, Lazarov O. Soluble amyloid precursor protein: a novel proliferation factor of adult progenitor cells of ectodermal and mesodermal origin. Stem Cell Res Ther. 2011;2(4):36. doi:10.1186/scrt77 21878106 PMC3219067

[19] Demars MP, Hollands C, Zhao KDT, Lazarov O. Soluble amyloid precursor protein-α rescues age-linked decline in neural progenitor cell proliferation. Neurobiol Aging. 2013;34(10):2431–2440. doi:10.1016/j.neurobiolaging.2013.04.016 23683827 PMC3706568

[20] Shabani K, Pigeon J, Benaissa Touil Zariouh M, The temporal balance between self-renewal and differentiation of human neural stem cells requires the amyloid precursor protein. Sci Adv. 2023;9(24):eadd5002. doi:10.1126/sciadv.add5002 37327344 PMC10275593

[21] Ovchinnikov DA, Korn O, Virshup I, Wells CA, Wolvetang EJ. The Impact of APP on Alzheimer-like Pathogenesis and Gene Expression in Down Syndrome iPSC-Derived Neurons. Stem Cell Rep. 2018;11(1):32–42. doi:10.1016/j.stemcr.2018.05.004 PMC6066957 29861166

[22] Sosa LJ, Postma NL, Estrada-Bernal A, Dosage of amyloid precursor protein affects axonal contact guidance in Down syndrome. FASEB J Off Publ Fed Am Soc Exp Biol. 2014;28(1):195–205. doi:10.1096/fj.13-232686 PMC3868833 24036883

[23] Selkoe DJ. Alzheimer’s disease: genes, proteins, and therapy. Physiol Rev. 2001;81(2):741–766. doi:10.1152/physrev.2001.81.2.741 11274343

[24] Maure-Blesa L, Carmona-Iragui M, Lott I, The history of Down syndrome-associated Alzheimer’s disease; past, present, and future. Alzheimers Dement J Alzheimers Assoc. 2025;21(6):e70158. doi:10.1002/alz.70158 PMC12138279 40469048

[25] Zigman WB, Lott IT. Alzheimer’s disease in Down syndrome: Neurobiology and risk. Ment Retard Dev Disabil Res Rev. 2007;13(3):237–246. doi:10.1002/mrdd.20163 17910085

[26] Ovchinnikov DA, Korn O, Virshup I, Wells CA, Wolvetang EJ. The Impact of APP on Alzheimer-like Pathogenesis and Gene Expression in Down Syndrome iPSC-Derived Neurons. Stem Cell Rep. 2018;11(1):32–42. doi:10.1016/j.stemcr.2018.05.004 PMC6066957 29861166

[27] Wu CI, Vinton EA, Pearse RV, APP and DYRK1A regulate axonal and synaptic vesicle protein networks and mediate Alzheimer’s pathology in trisomy 21 neurons. Mol Psychiatry. 2022;27(4):1970–1989. doi:10.1038/s41380-022-01454-5 35194165 PMC9133025

[28] Lu HE, Yang YC, Chen SM, Modeling neurogenesis impairment in Down syndrome with induced pluripotent stem cells from Trisomy 21 amniotic fluid cells. Exp Cell Res. 2013;319(4):498–505. doi:10.1016/j.yexcr.2012.09.017 23041301

[29] Utagawa EC, Moreno DG, Schafernak KT, Neurogenesis and neuronal differentiation in the postnatal frontal cortex in Down syndrome. Acta Neuropathol Commun. 2022;10(1):86. doi:10.1186/s40478-022-01385-w 35676735 PMC9175369

[30] Hernández-Ortega K, Canul-Euan AA, Solis-Paredes JM, S100B actions on glial and neuronal cells in the developing brain: an overview. Front Neurosci. 2024;18. doi:10.3389/fnins.2024.1425525 PMC11256909 39027325

[31] Shapiro LA, Bialowas-McGoey LA, Whitaker-Azmitia PM. Effects of S100B on Serotonergic Plasticity and Neuroinflammation in the Hippocampus in Down Syndrome and Alzheimer’s Disease: Studies in an S100B Overexpressing Mouse Model. Cardiovasc Psychiatry Neurol. 2010;2010:153657. doi:10.1155/2010/153657 PMC2933893 20827311

[32] Matsui Lee IS, Suzuki M, Hayashi N, Copper-dependent formation of disulfide-linked dimer of S100B protein. Arch Biochem Biophys. 2000;374(2):137–141. doi:10.1006/abbi.1999.1595 10666291

[33] Thulin E, Kesvatera T, Linse S. Molecular Determinants of S100B Oligomer Formation. PLOS ONE. 2011;6(3):e14768. doi:10.1371/journal.pone.0014768 21445240 PMC3060798

[34] Langeh U, Singh S. Targeting S100B Protein as a Surrogate Biomarker and its Role in Various Neurological Disorders. Curr Neuropharmacol. 2021;19(2):265–277. doi:10.2174/1570159X18666200729100427 32727332 PMC8033985

[35] Lu J, Esposito G, Scuderi C, S100B and APP Promote a Gliocentric Shift and Impaired Neurogenesis in Down Syndrome Neural Progenitors. PLOS ONE. 2011;6(7):e22126. doi:10.1371/journal.pone.0022126 21779383 PMC3133657

[36] Stagni F, Bartesaghi R. The Challenging Pathway of Treatment for Neurogenesis Impairment in Down Syndrome: Achievements and Perspectives. Front Cell Neurosci. 2022;16:903729.doi:10.3389/fncel.2022.903729 35634470 PMC9130961

[37] Shabani K, Pigeon J, Benaissa Touil Zariouh M, The temporal balance between self-renewal and differentiation of human neural stem cells requires the amyloid precursor protein. Sci Adv. 9(24):eadd5002. doi:10.1126/sciadv.add5002 PMC10275593 37327344

[38] Chen XQ. A pilot exploration with Posiphen to normalize amyloid precursor protein in Down syndrome. Neural Regen Res. 2021;16(12):2420–2421. doi:10.4103/1673-5374.313044 33907026 PMC8374580

[39] Chen XQ, Salehi A, Pearn ML, Targeting increased levels of APP in Down syndrome: Posiphen-mediated reductions in APP and its products reverse endosomal phenotypes in the Ts65Dn mouse model. Alzheimers Dement J Alzheimers Assoc. 2021;17(2):271–292. doi:10.1002/alz.12185 PMC7984396 32975365

[40] Ryoo SR, Jeong HK, Radnaabazar C, DYRK1A-mediated Hyperphosphorylation of Tau. J Biol Chem. 2007;282(48):34850–34857. doi:10.1074/jbc.M707358200 17906291

[41] Duchon A, Herault Y. DYRK1A, a Dosage-Sensitive Gene Involved in Neurodevelopmental Disorders, Is a Target for Drug Development in Down Syndrome. Front Behav Neurosci. 2016;10:104. doi:10.3389/fnbeh.2016.00104 27375444 PMC4891327

[42] Griffin WST, Sheng JG, McKenzie JE, Life-long overexpression of S100β in Down’s syndrome: implications for Alzheimer pathogenesis. Neurobiol Aging. 1998;19(5):401–405. doi:10.1016/S0197-4580(98)00074-8 9880042 PMC3833593

[43] Guan T, Guo Y, Zhou T, Oxidized SOD1 accelerates cellular senescence in neural stem cells. Stem Cell Res Ther. 2024;15(1):55. doi:10.1186/s13287-024-03669-5 38414053 PMC10900543

[44] Taylor JM, Crack PJ. Impact of Oxidative Stress on Neuronal Survival. Clin Exp Pharmacol Physiol. 2004;31(7):397–406. doi:10.1111/j.1440-1681.2004.04017.x 15236624

[45] Chidambaram SB, Anand N, Varma SR, Superoxide dismutase and neurological disorders. IBRO Neurosci Rep. 2024;16:373–394. doi:10.1016/j.ibneur.2023.11.007 39007083 PMC11240301

