# Supplementary material for: Amyloid precursor protein dosage normalization rescues neurogenesis and Alzheimer’s Disease phenotypes associated with Down Syndrome
Source: Med Res Arch. Author manuscript; Available in PMC 2026 Sep 1. (PMC13529314; doi:10.18103/mra.2026.0302)
Supplement: Supplementary file 1 [file NIHMS2203834-supplement-1.pdf]

Table S1. Antibodies used in the experiments.

REAGENT or RESOURCE	SOURCE	CATALOGUE #
**Antibodies**		
Rabbit anti-DCX (IHC 1:200; WB 1:1000)	Abcam	ab18723
Mouse anti-beta-3 Tubulin (IHC 1:250; WB 1:1000)	Thermo Fischer Scientific	MA1-118
Rabbit anti-Sox2 (IHC 1:200; WB 1:1000)	Abcam	ab97959
Donkey anti-rabbit 488 (ICC/IHC 1:500)	Jackson ImmunoResearch Labs	711-545-152

REAGENT or RESOURCE	SOURCE	CATALOGUE #
Donkey anti-mouse 488 (ICC/IHC 1:500)	Jackson ImmunoResearch Labs	715-545-151
Donkey anti-rabbit Cy3 (ICC/IHC 1:500)	Jackson ImmunoResearch Labs	711-165-152
Donkey anti-rabbit Cy5 (ICC/IHC 1:500)	Jackson ImmunoResearch Labs	711-175-152
Donkey anti-mouse Cy5 (ICC/IHC 1:500)	Jackson ImmunoResearch Labs	715-175-151
Goat anti-mouse IgG-HRP (WB 1:10000)	Promega	W402B
Goat anti-rabbit IgG-HRP (WB 1:10000)	Promega	W4011
Mouse anti-NFH (WB 1:5000)	Proteintech	60331-1-Ig
Mouse GAPDH (WB 1:2500)	Thermo Fischer Scientific	MA5-15738
Rabbit anti-APP (WB 1:1000)	Millipore Sigma	A8717
Rabbit anti-BACE1 (WB 1:1000)	Cell signaling	5606S
Mouse anti-AT8 (WB 1:1000)	Thermo Fischer Scientific	MN1020
Mouse anti-Tau5 (WB 1:1000)	Thermo Fischer Scientific	AHB0042
Rabbit anti-DYRKIA (WB 1:1000)	Cell signaling	2771S
Rabbit anti-BACE2 (WB 1:1000)	Cell signaling	80357
Mouse anti-SOD1 (WB 1:1000)	Santa Cruz	sc-17767
Rabbit anti-S100β (WB 1:1000)	Abcam	ab52642
Mouse anti-β-Amyloid (6E10) (WB 1:1000)	BioLegend	803001
